# Bariatric surgery and cardiovascular outcome

**DOI:** 10.1186/s43044-020-00096-8

**Published:** 2020-10-02

**Authors:** Waleed Ammar, Hossam Abdel Basset, Amr AL Faramawy, Tarek Hegazy, Yasser Sharaf

**Affiliations:** 1 Department of Cardiology, Kasr Al Aini Hospital, Faculty of Medicine, Cairo University, Cairo, 11562 Egypt; 2 Department of Cardiology, Military Medical Academy, Cairo, Egypt; 3 Department of General Surgery, Faculty of Medicine, Cairo University, Cairo, 11562 Egypt

**Keywords:** Obesity, Bariatric surgery, Cardiovascular, Risk profile

## Abstract

**Background:**

Obesity is recognized as a classic risk factor for atherosclerosis and subsequent cardiovascular disease (CVD). Weight loss after bariatric surgery has been associated with reduced CV mortality and total mortality in obese patients. Our aim was to study the impact of bariatric surgery on CV risk profile, cardiac structure, and function postoperatively.

**Results:**

This prospective longitudinal study included 100 morbidly obese patients at final analysis. All patients were subjected to full clinical, laboratory, and echocardiographic examination at baseline and 6 months after bariatric surgery. The mean age of study population was 37.2 ± 10.49 with BMI of 47 ± 6.82. Females represented 84%. Sleeve gastrectomy and Roux-en-Y gastric bypass were performed in 79% and 21%, respectively. Surgery-related mortality and morbidity were 0.94% and 4.7%, respectively. After 6 months, there were significant decreases in BMI, heart rate, SBP, DBP, and Framingham risk score (*P* < 0.0001). The prevalence of risk factors decreased as follows: hypertension 24% vs. 12%, *P* = 0.0005; DM 21% vs. 11%, *P* = 0.002; dyslipidemia 32% vs. 7%, *P* < 0.0001; and metabolic syndrome 54% vs. 26%, *P* < 0.0001. Highly significant (*P* < 0.0001) decrease in fasting PG and 2 h PP-PG, HbA1c, ASL, ALT, fasting total cholesterol, LDL, TG, and increase in HDL were observed after bariatric surgery. There were significant shortening in QTc interval (*P* = 0.009), decrease in LV dimensions and LV mass index (*P* < 0.0001), and increase in LV EF% (*P* = 0.0003). BMI at follow-up showed significant positive correlation with age, Framingham risk score, and preoperative BMI (*r* = 0.289, *P* = 0.0036; *r* = 0.37, *P* = 0.0054; and *r* = 0.31, *P* = 0.0081, respectively).

**Conclusion:**

In addition to enabling patients to achieve a substantial weight loss, bariatric surgery provides a myriad of health benefits. Weight reduction was associated with a favorable improvement in cardiovascular risk profile, cardiac structure, and function.

## Background

Obesity has become a global epidemic and major problem in the twenty-first century which influences many aspects of health [1]. Obesity has a strong causal relationship with numerous serious comorbidities that impair quality of life, shorten life expectancy, and carry a major economic burden [2]. It is estimated that at least 2.8 million adults die each year due to obesity-related cardiovascular disease [3].

Behavioral changes and pharmacological treatment result in reduction of only 5 to 10% in body weight [4]. On the other hand, many studies have demonstrated that bariatric surgery is associated with significant and durable weight loss and improvement of obesity-related comorbidities [5, 6].

There are multiple options for the surgical management of morbid obesity in the appropriate candidate. The procedures can be characterized as restrictive procedures, sleeve gastrectomy (SG); mal-absorptive procedures, bilio-pancreatic diversion with duodenal switch; or combination procedures, Roux-en-Y gastric bypass (RYGB). Although these categorizations may be overly simplistic, the distinctions are useful. All of these operations are commonly performed using laparoscopic approaches.

Obesity, particularly severe obesity, is capable of producing hemodynamic alterations that contribute to changes in cardiac morphology which may predispose to impairment of ventricular function and heart failure. Substantial voluntary weight loss is capable of reversing many of the hemodynamic, neurohormonal, and metabolic alterations associated with obesity [7].

### Aim of the work

To assess the prevalence of cardiovascular risk factors in morbidly obese patients and to study the impact of bariatric surgery on cardiovascular (CV) risk factors, cardiac structure, and function postoperatively.

## Methods

This was a prospective longitudinal study that was conducted from January 2017 to April 2018 in Maadi Military Medical Compound and cardiology department, Cairo University. One hundred patients with body mass index (BMI) ≥ 35 kg/m^2^ aged between 18 and 70 years were included. Patients with any contraindication to surgery and those who refuse to participate in this study were excluded.

All patients had a thorough medical history and clinical examination with an emphasis on cardiac risk factors. The BMI was calculated as body weight (kg)/the square of the height (m^2^), and waist circumference was measured in inches.

- Laboratory workup included fasting lipid profile, fasting and 2 h post-prandial plasma glucose, HbA1c, liver enzymes (AST and ALT), serum urea, and creatinine.
- Cardiac workup included 12 lead ECG and full echocardiographic study using (vivid 7; GE Healthcare, Horten, Norway) machine with GE S3 probe. All two-dimensional guided M-mode echocardiographic measurements were taken according to the American Society of Echocardiography and the European Association of Cardiovascular Imaging guidelines [8]. Left ventricular mass was measured by using Deveraux et al.’s equation, and the definition of left ventricular hypertrophy (LVH) in this study is left ventricular mass index (LVMI) ≥ 110 g/m^2^ in females and ≥ 125 g/m^2^ in males. Left ventricular ejection fraction was measured by using the Teichholz equation, and transmitral pulsed Doppler E and A wave was used to assess the diastolic function.

### Cardiovascular risk factors

*Hypertension* was defined as systolic BP (SBP) ≥ 140 mmHg, and/or diastolic BP (DBP) ≥ 90 mmHg, and/or the use of anti-hypertensive medications [9].

*Diabetes mellitus* was defined as a hemoglobin A1c (HbA1c) ≥ 6.5%, or fasting plasma glucose (FPG) ≥ 126 mg/dL, or a 2-h plasma glucose ≥ 200 mg/dL and/or the use of anti-diabetic agents [10].

*Dyslipidemia* was considered when low-density lipoprotein (LDL) cholesterol was ≥ 130 mg/dL, or high-density lipoprotein (HDL) cholesterol ≤ 50 mg/dL in women and ≤ 40 mg/dL in men, increased triglycerides (TGs) ≥ 150 mg/dL, or if the patient is currently receiving a lipid lowering agent [11].

*Metabolic syndrome*, according to the American Heart Association (AHA) and the National Heart, Lung, and Blood Institute (NHLBI) definition, is present if ≥ 3 of the following 5 criteria are met: waist circumference ≥ 40 in. (men) or 35 in. (women), blood pressure ≥ 130/85 mmHg, fasting TG ≥ 150 mg/dL, fasting HDL cholesterol < 40 mg/dL (men) or 50 mg/dL (women), and FPG ≥ 100 mg/dL [12].

*Framingham risk score* is a gender-specific algorithm used to estimate the 10-year cardiovascular risk of an individual [13].

*Estimated vascular age* is the chronological age of an individual adjusted by their level of atherosclerosis. It was calculated according to the definition of D’Agostino et al. in the tables from the Framingham Heart Study [13].

#### Follow-up

Patients were followed up for 6 months after surgery at which they were subjected to full clinical evaluation, laboratory workup, electrocardiogram, and full echocardiographic study.

### Ethical approval

The study was conducted in accordance with the Declaration of Helsinki and was approved by the Research Ethics Committee of the Faculty of Medicine (reference number I-150314). All study participants provided informed written consent.

### Statistical analysis

Data entry, processing, and statistical analysis were carried out using MedCalc ver. 18.2.1 (MedCalc, Ostend, Belgium).

#### Descriptive statistics

Mean, standard deviation (± SD), and range were used for parametric numerical data, while median and interquartile range (IQR) for non-parametric numerical data. Frequency and percentage was used for non-numerical data.

#### Analytical statistics

Mann-Whitney’s test (*U* test) was used to assess the statistical significance of the difference of a non-parametric variable between two study groups, Wilcoxon’s test for the difference of a non-parametric variable between two (paired) study group means, and repeated measures and factorial ANOVA tests for the difference between more than two (paired) study group means, with the ability to insert grouping factors, which was used to generate clustered multiple variable graphs. Chi-square test was used to examine the relationship between two qualitative variables. Correlation analysis (using Spearman’s method) was used to assess the strength of association between two quantitative variables. The correlation coefficient denoted symbolically as *r* defines the strength and direction of the linear relationship between two variables. Multiple linear regression was used to test and estimate the dependence of a quantitative variable based on its relationship with a set of independent variables, and logistic regression for the prediction of the presence or absence of an outcome based on a set of independent variables. It is similar to a linear regression model but is suited when the dependent variable is qualitative (categorical). *P* value < 0.05 (5%) was considered to be statistically significant, and *P* < 0.01 highly significant.

## Results

One hundred and six patients fulfilled the inclusion criteria and were initially recruited in the study, one of them died postoperatively, and five patients did not complete follow-up workup. One hundred patients completed the follow-up resulting in a follow-up rate of 94% and were included in the final analysis. The mean age of study population was 37.2 ± 10.49 with BMI of 47 ± 6.82. Females represented 84% and males 16%.

### Operative data

#### Type of surgery

Among the 100 patients who completed the follow-up analysis, sleeve gastrectomy was done in 79 patients (79%) whereas gastric bypass was done in 21 patients (21%).

#### Adverse surgical outcomes

One patient developed fatal pulmonary embolism 1 day after gastric bypass (GB) surgery. Three patients in the GB group developed anastomotic site leakage 2–4 days of the procedure that was managed surgically with a smooth course thereafter. Two patients in the GB group developed intestinal adhesions 3–4 months following surgery and were managed surgically.

### Impact of bariatric surgery on cardiovascular risk profile

The impact of the bariatric surgery on BMI, blood pressure, DM, dyslipidemia, and, consequently, metabolic syndrome, Framingham risk score, and estimated vascular age after 6 months of surgery is clearly shown in Table 1.

**Table 1:** Clinical characteristics preoperative and 6 months after bariatric surgery

Variables		Preoperative measurements, median (IQR)	6 months FU measurements, median (IQR)	*P* value^a^
BMI		45 (43.2–49.3)	32.8 (31.1–35.7)	< 0.0001**
Systolic BP		125 (120–132.5)	120 (115–130)	< 0.0001**
Diastolic BP		80 (70–85)	78 (70–80)	= 0.0002**
Estimated vascular age		51 (36–76)	42 (34–61)	< 0.0001**
Framingham risk score		5.3 (2–13.3)	2.8 (1.6–7.3)	< 0.0001**
Variables		Preoperative measurements	6 months FU measurements	*P* value^b^
Hypertension		24 (24%)	12 (12%)	= 0.0005**
Diabetes mellitus		21 (21%)	11 (11%)	= 0.002**
Dyslipidemia		32 (32%)	7 (7%)	< 0.0001**
Metabolic syndrome		54 (54%)	26 (26%)	< 0.0001**
Risk category (Framingham risk score)	Low	45 (61.6%)	57 (78.1%)	< 0.0001**
	Moderate	7 (9.6%)	4 (5.5%)	
	High	21 (28.8%)	12 (16.4%)	

*BMI* Body Mass index, *BP* Blood pressure, *IQR* Interquartile range

^a^Using Wilcoxon’s test

^b^Using chi-square test

As shown in Table 2, 6 months after the bariatric surgery, there was a considerable improvement of all lipid sub-fractions and significant reduction in FPG, 2 h PP-PG, HbA1c, and liver enzymes (*P* < 0.0001).

**Table 2:** Laboratory workup preoperative and 6 months after bariatric surgery

Variables	Preoperative measurements, median (IQR)	6 months FU measurements, median (IQR)	*P* value^a^
Total cholesterol (mg/dL)	195.5 (168–240)	170 (150–200)	< 0.0001**
TGs (mg/dL)	102 (80–156.5)	90.5 (73.5–120)	< 0.0001**
LDL (mg/dL)	100 (86–120)	94 (82–100)	< 0.0001**
HDL (mg/dL)	51 (47.5–57)	54 (50.5–59)	< 0.0001**
FPG (mg/dL)	84 (75–111.5)	78 (72.5–100)	< 0.0001**
PP-PG (mg/dL)	115.5 (103–142)	110 (102–126)	< 0.0001**
HbA1C (mg/dL)	5.1 (4.9–5.9)	5 (4.8–5.6)	< 0.0001**
ALT (U/L)	29 (22.5–44.5)	25.5 (20.5–36.5)	< 0.0001**
AST (U/L)	29 (20.5–43)	25 (17–32.5)	< 0.0001**
Creatinine (mg/dL)	0.7 (0.6–0.8)	0.7 (0.6–0.7)	= 0.146
Urea (mg/dL)	24 (19–34)	20 (17–27)	< 0.0001**

*ALT* Alanine aminotransferase, *AST* Aspartate aminotransferase, *FPG* Fasting plasma glucose, *HbA1c* Glycosylated hemoglobin, *HDL* High-density lipoprotein, *IQR* interquartile range, *LDL* Low-density lipoprotein, *PP-PG* Post-prandial plasma glucose, *TGs* Triglycerides

^a^Using Wilcoxon’s test

### Impact of bariatric surgery on electrocardiographic and echocardiographic measurements

It is notable in Table 3 that weight loss with bariatric surgery resulted in significant reduction in resting heart rate and shortening in corrected QT interval as a marker of ventricular repolarization.

**Table 3:** Electrocardiographic findings preoperative and 6 months after bariatric surgery

Variables	Preoperative measurement, median (IQR)	6 months FU measurements, median (IQR)	*P* value^a^
Heart rate (bpm)	82 (75–92)	76 (71–82)	< 0.0001**
PR interval (msec)	160 (140–160)	160 (150–162)	= 0.354
QRS complex width (msec)	80 (80–100)	80 (80–100)	= 1.000
QTc interval (msec)	432 (416–447)	423 (409–447)	= 0.0094**

*bpm* Beats per minute, *IQR* Interquartile range, *msec* Milliseconds

^a^Using Wilcoxon’s test

Echocardiographic findings at 6 months follow-up revealed significant reduction in LV dimensions and LV mass index (*P* < 0.0001). In Table 4, it is notable that there was a significant increase in LV EF% (*P* = 0.0003) and increase in E/A ratio (*P* < 0.0001).

**Table 4:** Echocardiographic measurements preoperative and 6 months after bariatric surgery

Variables	Preoperative measurements, median (IQR)	6 months FU measurements, median (IQR)	*P* value^a^
**LA** (cm)	3.2 (2.9–3.7)	3.2 (2.9–3.7)	= 1.000
**AO** (cm)	3.05 (2.8–3.45)	3 (2.8–3.45)	= 0.273
**LVEDD** (cm)	4.95 (4.6–5.4)	4.9 (4.5–5.3)	< 0.0001**
**LVESD** (cm)	3.1 (2.7–3.5)	3 (2.7–3.4)	< 0.0001**
**IVSD** (cm)	1 (0.8–1.2)	1 (0.8–1)	= 0.735
**PWD** (cm)	1 (0.8–1.1)	0.99 (0.8–1.1)	= 0.441
**FS** (%)	36 (33–40)	37.5 (34–40)	< 0.0001**
**EF** (%)	65 (62–69)	67 (63–69)	= 0.0003**
**LVMI** (g/m^2^)	47.7 (32.2–59.2)	40.5 (29.4–51.1)	< 0.0001**
**E wave** (m/s)	0.8 (0.7–0.9)	0.8 (0.7–0.92)	= 0.365
**A wave** (m/s)	0.5 (0.5–0.65)	0.5 (0.5–0.6)	= 0.401
**E/A ratio**	1.5 (1.2–1.8)	1.6 (1.5–1.8)	< 0.0001**

*AO* Aortic root, *EF* ejection fraction, *FS* Fractional shortening, *IQR* Interquartile range, *IVSD* Interventricular septal dimension, *LVEDD* Left ventricular end diastolic dimension, *LVESD* Left ventricular end systolic dimension, *LVMI* Left ventricular mass index, *PWD* Posterior wall dimension

^a^Using Wilcoxon’s test

### Correlation studies

**Fig. 1:**
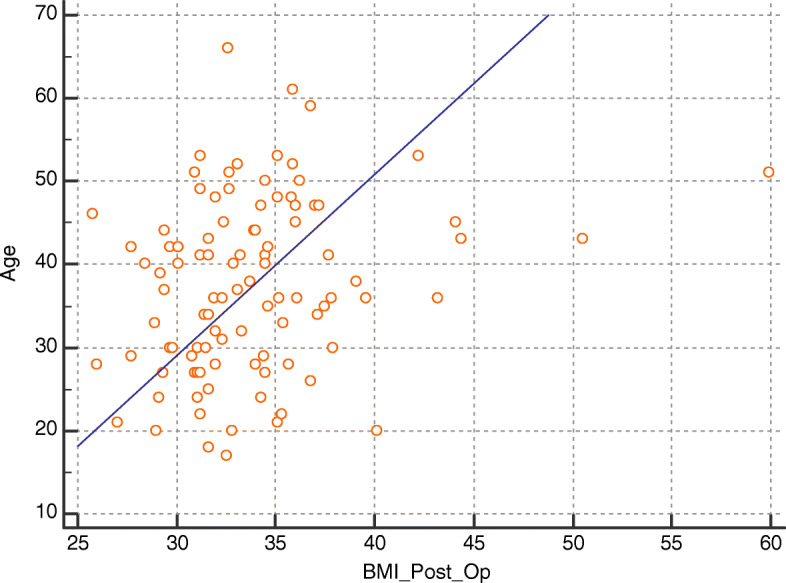
Correlation between postoperative BMI and age

**Fig. 2:**
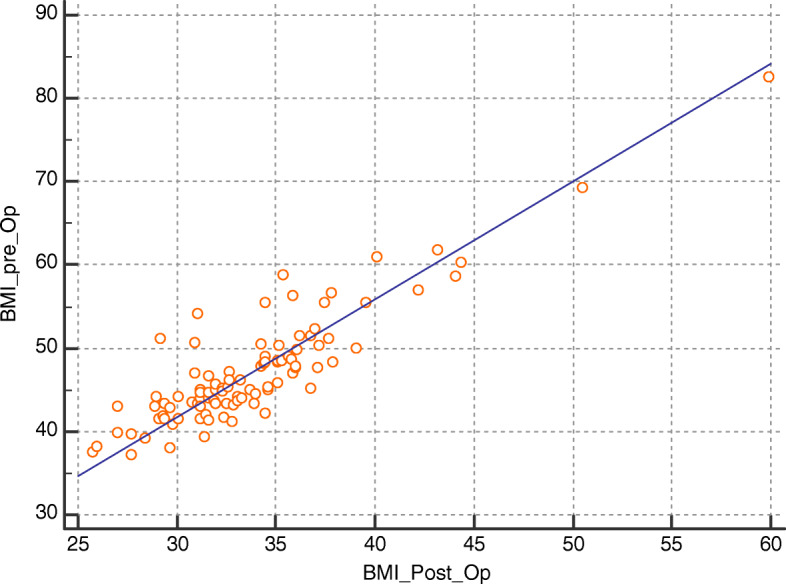
Correlation between postoperative BMI and preoperative BMI

**Fig. 3:**
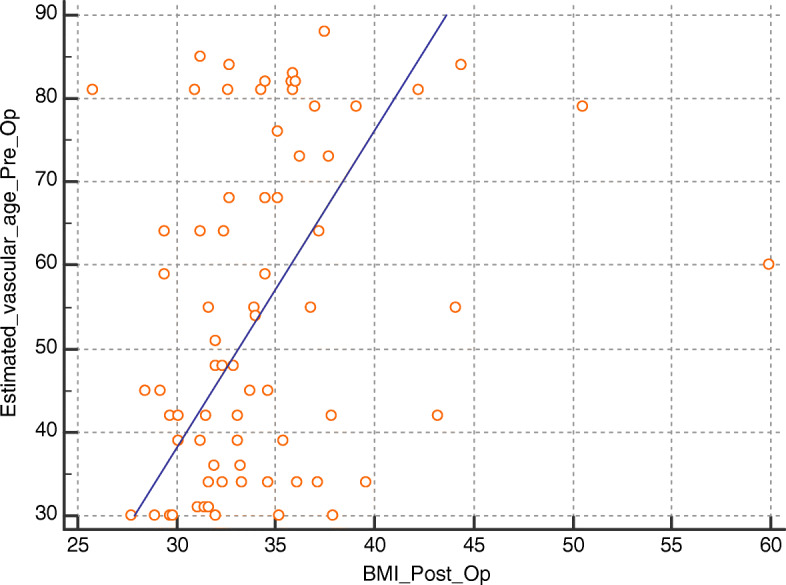
Correlation between postoperative BMI and preoperative estimated vascular age

**Fig 4:**
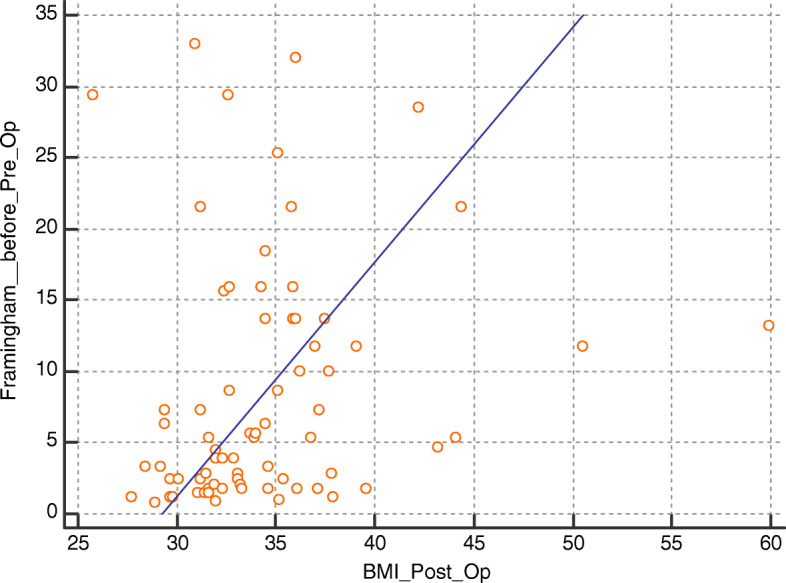
Correlation between postoperative BMI and preoperative Framingham score

Using Spearman’s correlation coefficient, there has been a statistically significant positive correlation between BMI at 6 months follow-up and clinical variables including age, heart rate, vascular age, Framingham score, and preoperative BMI (*r* = 0.289, *P* = 0.0036; *r* = 0.24, *P* = 0.015; *r* = 0.79, *P* < 0.0001; *r* = 0.37, *P* = 0.0054; and *r* = 0.31, *P* = 0.0081, respectively). Such correlations are shown in Figs. 1, 2, 3, and 4.

Spearman’s correlation analysis showed that preoperative echocardiographic findings, LA, AO, LVESD, IVSD, PWD, LV mass index, and A wave, had a highly significant positive correlation with 6 months postoperative BMI, whereas E wave and E/A ratio had a highly significant negative correlation with 6 months postoperative BMI (Table 5). Finally, multiple regression model using forward method revealed significant positive correlation (age and preoperative BMI) and significant negative correlation (preoperative EF) with postoperative BMI (Table 6).

**Table 5:** Correlations of echocardiographic data and postoperative BMI

	*r*	*P* value^a^
LA (cm)	0.223	= 0.025*
AO (cm)	0.288	= 0.0037**
LVEDD (cm)	0.183	= 0.0683
LVESD (cm)	0.213	= 0.033*
IVSD (cm)	0.450	< 0.0001**
PWD (cm)	0.273	= 0.0061**
FS (%)	− 0.170	= 0.0917
EF (%)	− 0.172	= 0.0864
LV MI (g/m^2^)	0.355	= 0.0003**
E wave (m/s)	− 0.316	= 0.0014**
A wave (m/s)	0.258	= 0.0097**
E/A ratio	− 0.297	= 0.0027**

*AO* Aortic root, *EF* Ejection fraction, *FS* fractional shortening, *IVSD* Interventricular septal dimension, *LVEDD* Left ventricular end diastolic dimension, *LVESD* Left ventricular end systolic dimension, *LVMI* Left ventricular mass index, *PWD* Posterior wall dimension

^a^Using Spearman’s correlation coefficient

**Table 6:** Multiple regression model for the factors affecting postoperative BMI

Predictor factor	***β***	SE	***P*** value
Constant	− 5.7961		
Age	0.06057	0.02102	0.0049**
BMI (pre-op)	0.6408	0.03352	< 0.0001**
EF% (pre-op)	− 0.1102	0.04636	0.019*

*BMI* Body mass index, *EF* Ejection fraction, *β* Regression coefficient, *SE* Standard error; using forward method

## Discussion

This was a prospective longitudinal trial; the mean age of study population was 37.2 ± 10.49 years. The majority (84%) of patients were females. These demographic characteristics were consistent with previous studies. In a meta-analysis involving 134 studies and an aggregate of 22,094 patients, 73% of them were females, with an average age of 39 years [14]. As in the current study, the population undergoing bariatric surgery is predominantly female in the fourth decade of life, probably when the metabolic and hemodynamic complications resulting from excess body weight appear from the clinical point of view. It is worth mentioning that, in our study, age was among the most important variables affecting BMI at follow-up that signify more expected weight loss when having surgery at younger age.

In our series, surgery-related mortality and morbidity were 0.9% and 4.7%, respectively. This remarkable accomplishment is primarily due to the introduction of laparoscopic techniques and a long-standing emphasis on safety and quality improvement [15]. In the American College of Surgeons Bariatric Surgery Network database, mortality 30 days after sleeve gastrectomy was 0.11% and Roux-en-Y gastric bypass 0.14%. The 30-day morbidity rate was 5.6% for sleeve gastrectomy and 5.9% for Roux-en-Y gastric bypass [16].

Regarding cardiovascular risk profile, the prevalence of hypertension was 21% with mean systolic and diastolic BP of 127 ± 13.5 and 80 ± 9.5 mmHg respectively. Dyslipidemia was evident in 32% of our patients, and the prevalence of DM was 24% with mean FBS and HbA1c of 99.8 ± 36.8 mg/dL and 5.6 ± 1.1%, respectively.

The prevalence of metabolic syndrome at baseline was 54%. This prevalence was low compared to other studies [17, 18], and this may be explained, at least in part, by the relatively low prevalence of dyslipidemia and hypertension in our study cohort. According to the Framingham risk score to estimate the risk of fatal or nonfatal coronary events in 10 years, risk categories in the current study were low (61.6%), intermediate (9.6%), and high (28.8%). Consistent with our results, in baseline analysis of the recent Traditional Brazilian Diet Trial including 150 adult patients, 55.3% of the study participants were classified as low risk, 4.7% as intermediate risk, and 40.0% as high 10-year CHD risk [19].

Bariatric surgery was associated with significant decrease in BMI after 6 months (45 (43.2–49.3) vs. 32.8 (31.1–35.7) with *P* value < 0.0001). This was in agreement with previous studies. De La Cruz-Muñoz et al. [20] studied the effectiveness of bariatric surgery in 71 patients. After 1 year, BMI was reduced from 49.7 to 39.2 kg/m^2^ among males and from 45.1 to 34.4 kg/m^2^ among females. In another study, Hady et al. [21] studied the impact of laparoscopic sleeve gastrectomy in 100 obese patients with 6 months follow-up period and reported significant reduction of BMI from 52.15 ± 8.5 to 37.98 ± 4.97 kg/m^2^.

Bariatric surgery was associated with significant improvement in various cardiovascular risk factors after 6 months in our study. The prevalence of hypertension decreased from 21 to 11% (47% remission) with highly significant decrease in systolic blood pressure (*P* < 0.0001) and diastolic blood pressure (*P* = 0.0002). In a study by Zhang et al. [22] with a total of 558 patients who underwent either LSG (200) or RYGB (358) for morbid obesity, the prevalence of hypertension was 52% at baseline, and after 6 months follow-up, there was 40% remission of hypertension. Additionally, in the systematic review of Heneghan et al., the prevalence of hypertension was 49% with 68% resolution or reduction in hypertension after a mean follow-up period of 34 months after bariatric surgery [23].

The prevalence of DM decreased from 24 to 12% (50% remission) and additional 16.6% reduction in needed medications. At Cleveland Clinic, 150 obese patients with T2DM were randomized to conventional medical therapy, RYGB, or SG. After 12 months, the primary endpoint which was the proportion of patients achieving HbA1c of 6.0% or less after treatment was achieved in 42% of the RYGB arm, 37% of the LSG arm, and 12% of the conventional medical therapy arm [24]. It is worth mentioning that the 2nd Diabetes Surgery Summit International Consensus Conference published recommendations that bariatric surgery should be considered as a treatment option for patients with T2D [25].

The prevalence of dyslipidemia decreased from 32 to 7% with resolution of dyslipidemia in 78% of our patients. Considerable improvement of all lipid sub-fractions was observed during follow-up in our study. This comes in concordance with Singhal et al. [26] who studied the effect of LSG on lipid profile of 50 obese patients. LSG resolved or improved lipid profile in a majority of patients during initial first 6 months after surgery. In another study, Strain et al. [27] studied 82 patients (67% female, age 46.4 ± 13.9) subjected to bariatric surgery. At 1 year, there was a significant reduction in triglycerides (*P* = 0.004) and significant increase in HDL (*P* = 0.025), while total cholesterol and LDL cholesterol showed no significant difference at follow-up. Furthermore, the systematic review of Heneghan et al. [23] came up with concordant results showing 71% resolution or reduction in dyslipidemia.

The prevalence of metabolic syndrome decreased from 54 to 26% at 6 months after bariatric surgery with 52% reduction in its prevalence. Batsis et al. [18] performed a population-based, retrospective study, in which bariatric surgery resulted in 67% reduction in the prevalence of metabolic syndrome. The number of patients with metabolic syndrome decreased from 156 (87%) to 53 (29%) after a mean follow-up of 3.4 years. Silva et al. [17] performed a prospective observational study composed of 96 patients with obesity, among which 86 were women, aged between 18 and 58 years old. At the end of 6 months, bariatric surgery resulted in 80% reduction in the prevalence of metabolic syndrome (69% vs. 14%, *P* < 0.0001).

At 6 months follow-up, there was a significant decrease in the estimated risk of fatal or nonfatal coronary events in 10 years according to the Framingham risk score (*P* < 0.0001) and estimated vascular age (*P* < 0.0001). In a recent study conducted by Blanco et al. [28] involving 360 patients with bariatric surgery, LSG was the most prevalent surgery (63%), followed by RYGB (20.6%), and reported significant reduction of both atherosclerotic cardiovascular disease and Framingham risk scores at 12 months. In another study, Wei et al. [29] recently investigated the benefit of CVD risk reduction after metabolic surgery in 392 obese patients with type 2 DM who had undergone LSG (87) or RYGB (305). The estimated 10-year coronary heart disease risk was reduced from 8.8 to 4.6% (*P* < 0.001). It is worth mentioning that our study further demonstrated statistically significant positive correlations between Framingham risk score, estimated vascular age, and postoperative 6-month BMI using Spearman’s correlation coefficient.

Electrocardiographic findings at 6 months follow-up revealed significant reduction in resting heart rate and shortening in QTc interval (*P* < 0.0001 and 0.009, respectively). Owan et al. [30] in the Utah Obesity Study included 423 severely obese patients undergoing bariatric surgery. At a 2-year follow-up, there was a large reduction in heart rate from 74 ± 12 to 60 ± 10. Omran et al. [31] performed a systematic review and meta-analysis of the effects of obesity and weight loss on the corrected QT interval. Weight loss was associated with a significant decrease in QTc (mean difference − 25.77 ms, 95% CI − 28.33–23.21). In another study, Mukerji et al. [32] investigated the effect of weight loss on ventricular repolarization in 39 normotensive morbidly obese patients. Again, weight loss was associated with significant reductions in mean QTc (from 428.7 ± 18.5 to 410.5 ± 11.9 ms, *P* < 0.0001). LV hypertrophy was a key determinant of QTc interval, and regression of LV hypertrophy related to weight loss was associated with shortening of QTc interval.

Bariatric surgery has been noted to induce changes in heart geometry and function, both systolic and diastolic. In our study, echocardiographic findings at 6 months follow-up revealed significant reduction in LV dimensions and LV mass index (*P* < 0.0001), increase in LV EF% (*P* = 0.0003), and increase in E/A ratio (*P* < 0.0001). Mauricio et al. [33] assessed the effect of BS in 41 patients, and there was a significant reduction in LVMI (101.3 ± 38.34 vs. 86.70 ± 26.65, *P* = 0.005) and increase in LV shortening fraction (31.05 ± 8.82% vs. 36.34 ± 8.21%, *P* = 0.007). Aggarwal et al. [34], in their systematic review and meta-analysis of the effect of bariatric surgery on cardiac structure and function, reported significant decrease of both left ventricular mass (mean decrease of 30 g) and mass index (mean decrease of 11%) in addition to significant decrease in LV end diastolic and systolic volumes and significant improvement in ejection fraction. Improvement of diastolic function postsurgical weight loss was also demonstrated by some other studies [35, 36]. It has been postulated that perivascular and interstitial LV fibrosis may contribute to LV diastolic dysfunction in obesity [37]. Our study sheds light on another potential and clinically relevant correlations; postoperative BMI showed significant correlation with LV mass index using Spearman’s correlation and with EF% using multiple regression model.

## Conclusion

Accumulating evidence supports our findings of significant cardiovascular risk reduction after bariatric surgery. Bariatric surgeries are reasonably safe procedures, and in addition to enabling patients to achieve a substantial weight loss, bariatric surgeries provide a myriad of health benefits. Major cardiovascular risk factors namely hypertension, diabetes mellitus, and dyslipidemia were reduced by 47%, 50%, and 78%, respectively. Additionally, the prevalence of metabolic syndrome, severity of Framingham risk score, and estimated vascular age were significantly improved after surgery. It should also be emphasized that a significant decrease in LV dimensions and LV mass index together with significant increase in LV ejection fraction was achieved at follow-up.

## Limitations

The findings from our study have to be considered in the context of some study limitations as well. First, there is a relatively short-term follow-up period after surgery. Second, personal differences in behavior, lifestyle, and treatment adherence postoperatively may have contributed to some extent in the observed outcome. Third, there is a lack of a control group. However, methodological methods are comparable to other recently published studies.

## Data Availability

The datasets used and/or analyzed during the current study are available from the corresponding author on reasonable request.

## Abbreviations

ALT: Alanine aminotransferase
AST: Aspartate aminotransferase
BMI: Body mass index
BS: Bariatric surgery
CHD: Coronary heart disease
CVD: Cardiovascular disease
DM: Diabetes mellitus
EF: Ejection fraction
FPG: Fasting plasma glucose
FS: Fractional shortening
HDL: High-density lipoprotein
LA: Left atrium
LDL: Low-density lipoprotein
LVEDD: Left ventricular end diastolic dimension
LVESD: Left ventricular end systolic dimension
PP-PG: Post-prandial plasma glucose
PWD: Posterior wall dimension
RYGB: Roux-en-Y gastric bypass
SG: Sleeve gastrectomy
IVSD: Interventricular septal dimension
TGs: Triglycerides
TTE: Transthoracic echocardiography
